# P-734. Heterosexual Syphilis in RI - Rising by Identity of Partner not Patient

**DOI:** 10.1093/ofid/ofaf695.945

**Published:** 2026-01-11

**Authors:** Maya Tsao-Wu, Joshua Tanzer, Alexi Almonte, Phillip Chan

**Affiliations:** Brown Internal Medicine, Providence, RI; Brown University Health, Providence, Rhode Island; Brown University Health, Providence, Rhode Island; Brown University Health, Providence, Rhode Island

## Abstract

**Background:**

Heterosexual syphilis is on the rise. While MSM persons still compose the majority of total cases in developed countries, their rate of growth is now dwarfed by non-MSM populations. In 2017-2021 syphilis rates increased 147% in MSW but only 8% in MSM, and in 2012-2021 syphilis in women 15 to 44 years of age rose 676%. The heterosexual epidemic is worrisome because it brings congenital syphilis (755% rise 2012-2021). Fetal outcomes for untreated congenital syphilis are approximately ⅓ ante/perinatal death, ⅓ clinically evident syphilis, and ⅓ asymptomatic, hopefully to be diagnosed later. These outcomes are preventable with appropriate antenatal screening and treatment, but recent data indicates our traditional screening is insufficient. Typical screening occurs at the first prenatal visit with additional screening if a mother is “high risk.” National data 2012-2016 showed zero traditional risk factors in nearly half of pregnant women with syphilis. Our project sought to identify traits that raise risk of syphilis for reproductive age heterosexual persons to inform clinician screening practices and contribute to the fight against congenital syphilis.

**Figure:**
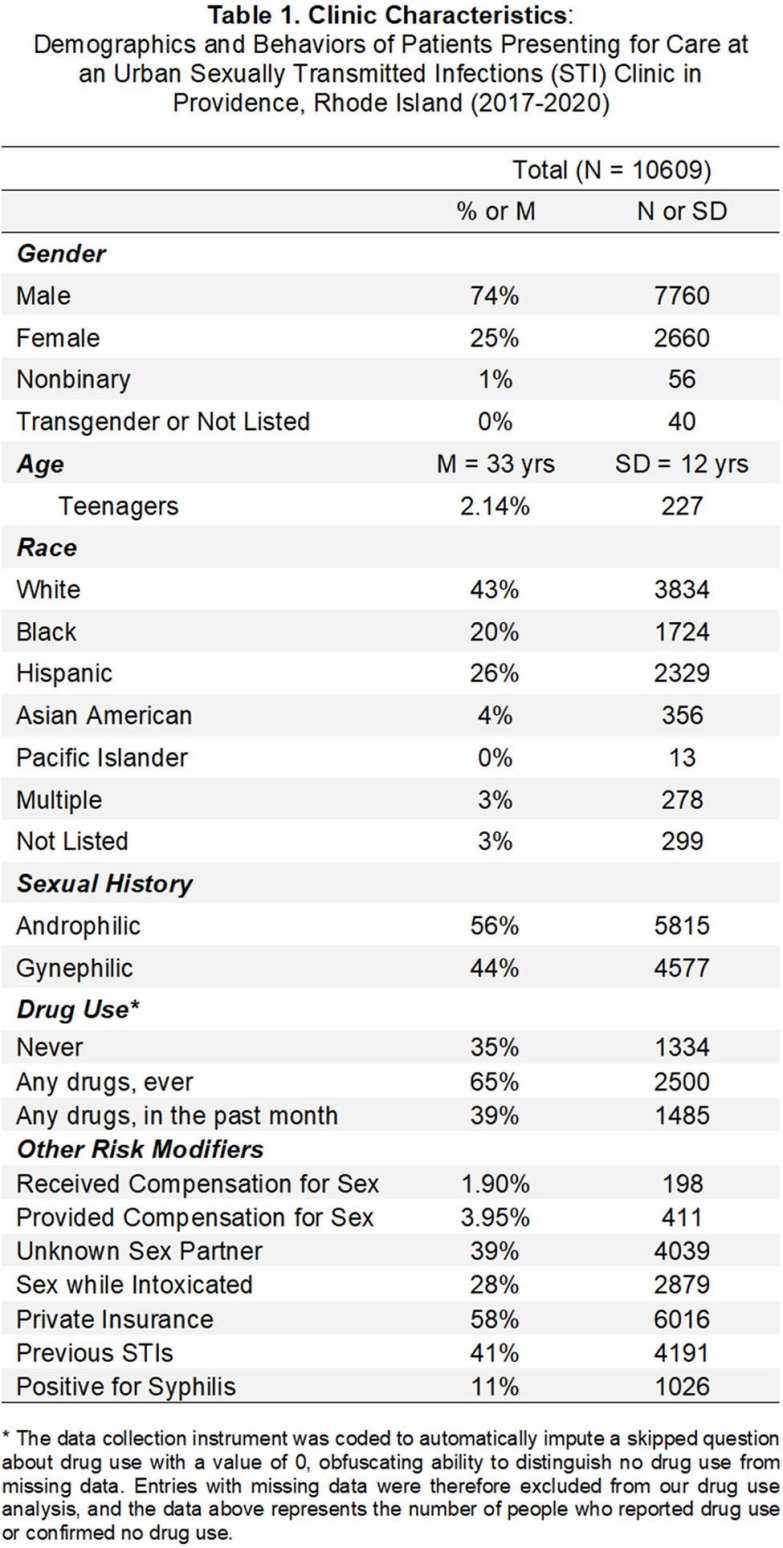

**Figure:**
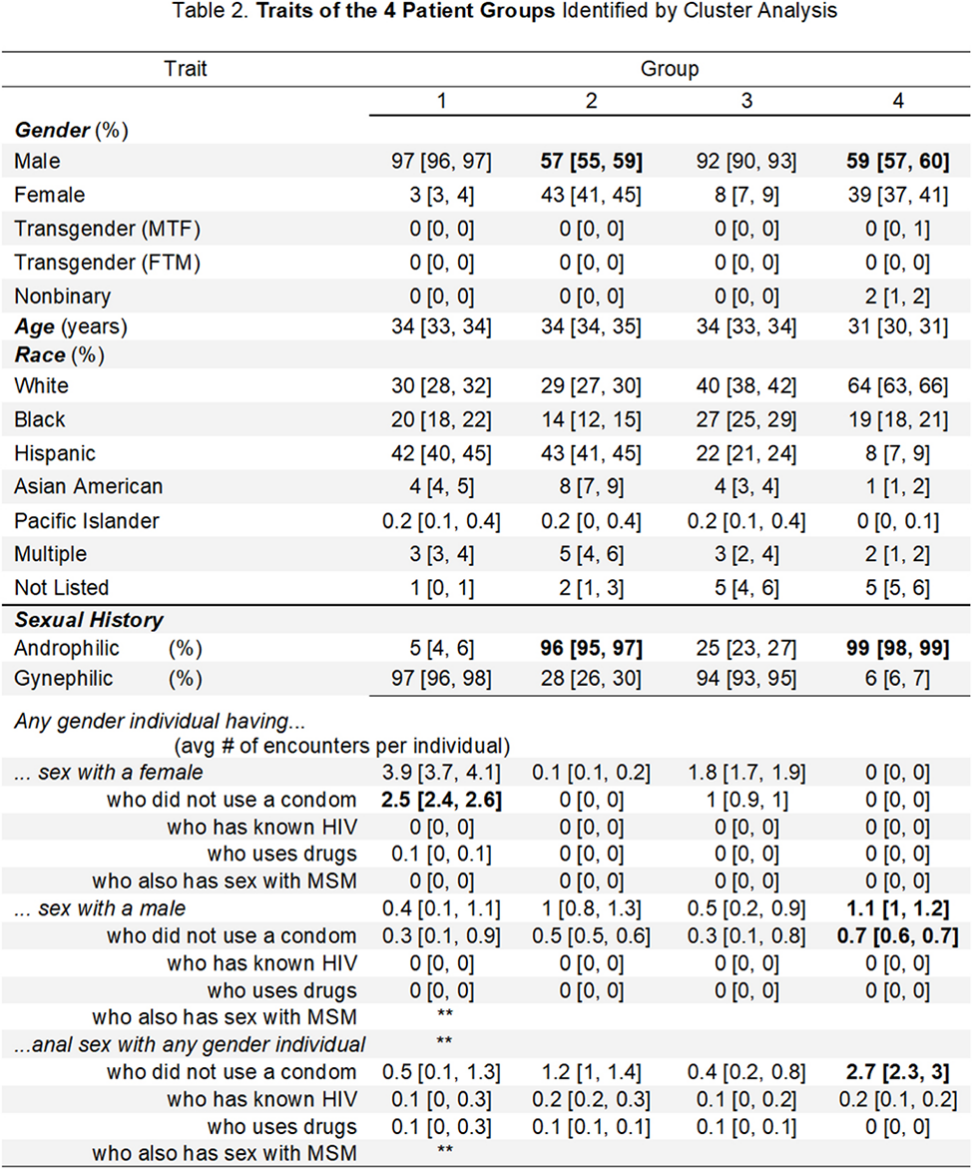

**Methods:**

We did secondary analysis of a prior dataset compiled from intake surveys of 10,609 patients who established care 2017-2020 at the main STI clinic in Rhode Island. Cluster analysis yielded four groups for whom we developed weighted risk scores from their demographic and behavioral traits based on national trends. We then compared our hypothesized risk scores to the groups' actual syphilis rates.

**Figure:**
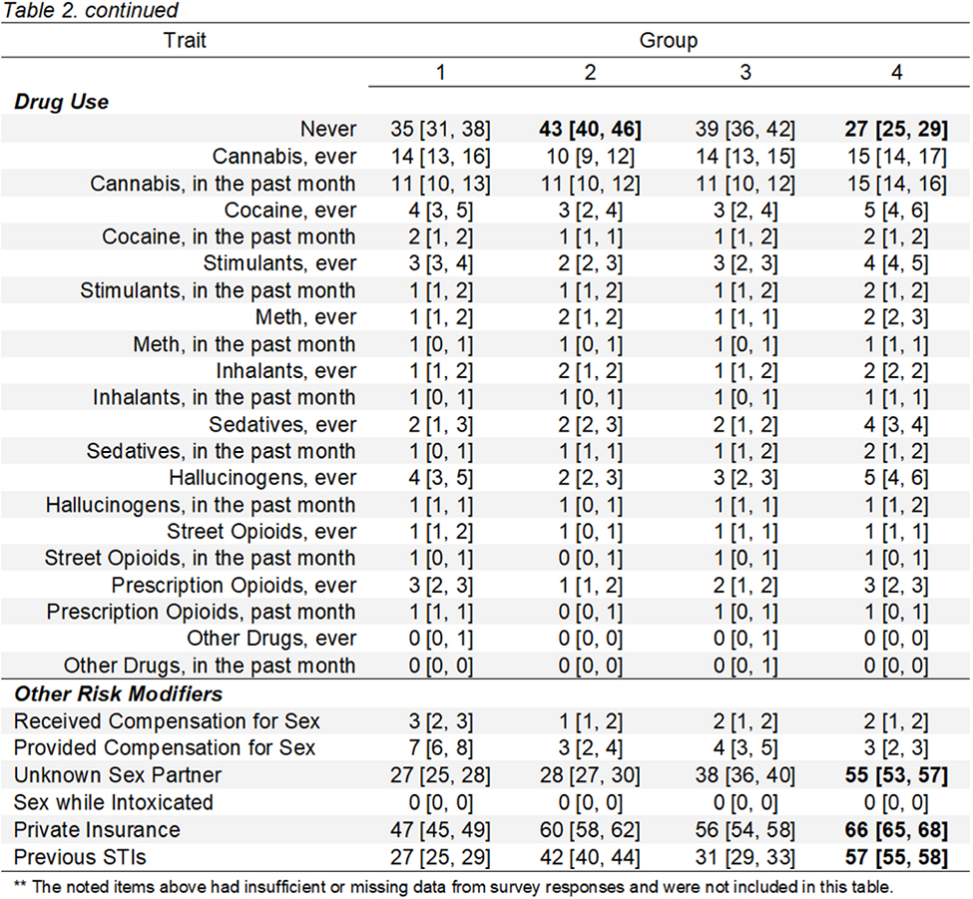

**Figure:**
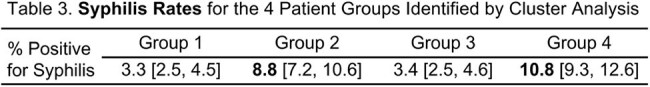

**Results:**

Syphilis rates from high to low were as follows: group 4 >2 > >1&3. Secondary traits were included in the tables below, but the key feature of groups 4&2 was androphilia, regardless of patient gender identity. Contrary to national trends and our expectations, drug use and race were not defining features of the high syphilis groups.

**Conclusion:**

Syphilis was associated not with MSM but with “SM," androphilia. For populations like that of our study, our data indicates that high-frequency syphilis screening should be ordered not only for “MSM identity” or other traditional high risk traits but for all patients whose partner is male.

**Disclosures:**

All Authors: No reported disclosures

